# *trans*-Bis(acetato-κ*O*)bis­(2-amino­ethanol-κ^2^ *N*,*O*)nickel(II)

**DOI:** 10.1107/S1600536812014237

**Published:** 2012-04-13

**Authors:** Mahdi Seifollahi Bazarjani, Sabine Foro, Wolfgang Donner, Aleksander Gurlo, Ralf Riedel

**Affiliations:** a Technische Universität Darmstadt, Fachbereich Material- und Geowissenschaften, Petersenstrasse 23, D-64287 Darmstadt, Germany

## Abstract

In the title compound, [Ni(CH_3_CO_2_)_2_(C_2_H_7_NO)_2_], the Ni^II^ cation, located on an inversion center, is *N*,*O*-chelated by two 2-amino­ethanol mol­ecules and further coordinated by two monodendate acetate anions in a slightly distorted octa­hedral geometry. The latter is stabilized by intra­molecular O—H⋯O hydrogen bonds involving the non-coordinated O atom of the acetate and the H atom of the hy­droxy group of the 2-amino­ethanol ligand. In the crystal, N—H⋯O hydrogen bonds link the mol­ecules into a three-dimensional supra­molecular framework that involves (*a*) the coordinated acetate O atom and one of the H atoms of the amino group and (*b*) the non-coordinated acetate O atom and the other H atom of the amino group.

## Related literature

For an application of the title compound, see: Baza­rjani *et al.* (2011 ▶). For the synthesis of NiO *via* the sol-gel route, see: Ozer & Lampert (1998 ▶); Livage & Ganguli (2001 ▶). For supra­molecular structures of transition metal complexes, see: Desiraju (1995 ▶, 2007 ▶). For related structures, see: Downie *et al.* (1971 ▶); Werner *et al.* (1996 ▶); Williams *et al.* (2001 ▶).
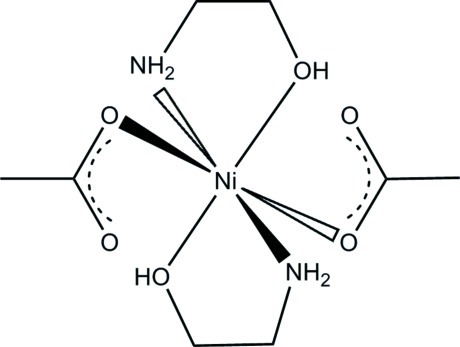


## Experimental

### 

#### Crystal data

[Ni(C_2_H_3_O_2_)_2_(C_2_H_7_NO)_2_]
*M* *_r_* = 298.97
Monoclinic, 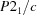

*a* = 5.3284 (5) Å
*b* = 9.216 (1) Å
*c* = 13.133 (2) Å
β = 94.22 (1)°
*V* = 643.17 (13) Å^3^
*Z* = 2
Mo *K*α radiation
μ = 1.53 mm^−1^
*T* = 293 K
0.16 × 0.08 × 0.06 mm

#### Data collection

Oxford Diffraction Xcalibur diffractometer with a Sapphire CCD detector
Absorption correction: multi-scan (*CrysAlis RED*; Oxford Diffraction, 2009 ▶) *T*_min_ = 0.792, *T*_max_ = 0.914
2309 measured reflections
1314 independent reflections
1035 reflections with *I* > 2σ(*I*)
*R*_int_ = 0.022

#### Refinement

*R*[*F* ^2^ > 2σ(*F* ^2^)] = 0.036
*wR*(*F* ^2^) = 0.093
*S* = 1.03
1314 reflections
89 parameters
3 restraints
H atoms treated by a mixture of independent and constrained refinement
Δρ_max_ = 0.32 e Å^−3^
Δρ_min_ = −0.25 e Å^−3^

### 

Data collection: *CrysAlis CCD* (Oxford Diffraction, 2009 ▶); cell refinement: *CrysAlis RED* (Oxford Diffraction, 2009 ▶); data reduction: *CrysAlis RED*; program(s) used to solve structure: *SHELXS97* (Sheldrick, 2008 ▶); program(s) used to refine structure: *SHELXL97* (Sheldrick, 2008 ▶); molecular graphics: *PLATON* (Spek, 2009 ▶); software used to prepare material for publication: *publCIF* (Westrip, 2010 ▶).

## Supplementary Material

Crystal structure: contains datablock(s) I, global. DOI: 10.1107/S1600536812014237/zl2465sup1.cif

Structure factors: contains datablock(s) I. DOI: 10.1107/S1600536812014237/zl2465Isup2.hkl

Additional supplementary materials:  crystallographic information; 3D view; checkCIF report

## Figures and Tables

**Table 1:** Hydrogen-bond geometry (Å, °)

*D*—H⋯*A*	*D*—H	H⋯*A*	*D*⋯*A*	*D*—H⋯*A*
N1—H11*N*⋯O2^i^	0.85 (2)	2.24 (2)	3.071 (3)	168 (3)
N1—H12*N*⋯O3^ii^	0.86 (2)	2.60 (2)	3.352 (3)	146 (3)
O1—H1*O*⋯O3	0.81 (2)	1.80 (2)	2.587 (3)	166 (3)

Symmetry codes: (i) 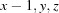; (ii) 
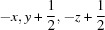
.

**Fig. 1.**
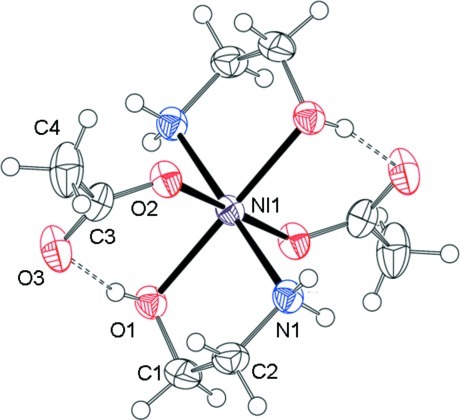
Molecular structure of the title compound. Displacement ellipsoids are shown at the 50% probability level.

**Fig. 2.**
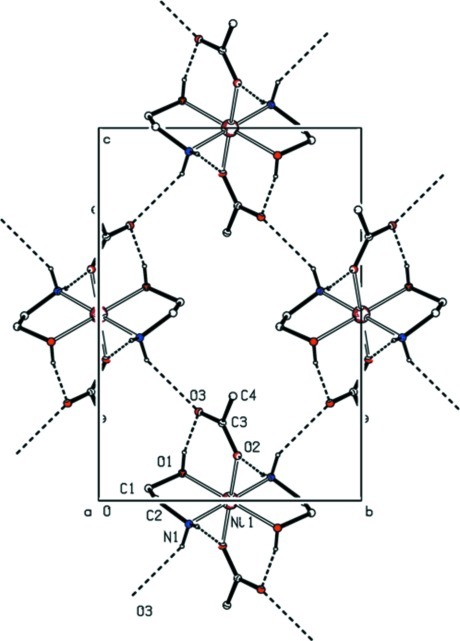
A perspective view of crystal structure of the title compound: intramolecular and intermolecular hydrogen bonding among the [Ni(CH3CO2)2(C2H7NO)2] units.

**Fig. 3.**
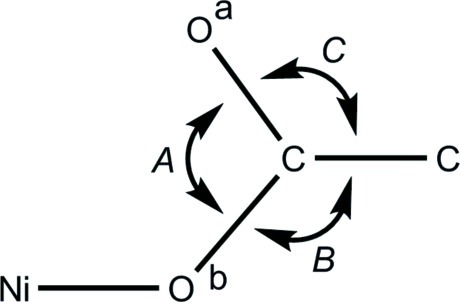
Geometry of the monodentate acetate group. For values of bond lengths a and b and bond angles A, B and C see Table 2.

## supplementary crystallographic information

### Comment

The synthesis of the title compound is performed at room temperature under ambient conditions by substituting H_2_O of [Ni(CH_3_CO_2_)_2_(H_2_O)_4_] with 2-aminoethanol. As the title compound is water-free, stable under ambient conditions and well soluble in lower alcohols, it represents a cost effective precursor for the sol-gel synthesis of NiO-based nanostrutures. The latter are of interest for switchable automobile mirrors and smart windows (Ozer & Lampert, 1998). Another application of the title compound is the synthesis of nanocomposite materials; nickel-polysilazane materials with ultrasmall and well dispersed nickel nanoparticles were obtained at room temperature in the reaction between the title compound and a polysilazane (Bazarjani *et al.*, 2011). The title compound possesses significantly higher stability and higher solubility in lower alcohols when compared with a similar Ni^II^ complex coordinated by two *N*,*N*-dimethylaminoethanol molecules, [Ni(CH_3_CO_2_)_2_(C_4_H_11_NO)_2_], which is air-sensitive (Williams *et al.*, 2001). These differences are due to the –NH_2_ group of the 2-aminoethanol ligand which is in the solid state hydrogen bonded to neighbouring [Ni(CH_3_CO_2_)_2_(C_2_H_7_NO)_2_] units and in solution it can get involved in hydrogen bonding with lower alcohols. The former results in the increased stability of the title compound, the latter is responsible for higher solubility of the title compound in alcohols (*e.g.* for methanol, compare 0.18 mol l^-1^ for the title compund to 0.10 mol l^-1^ for [Ni(CH_3_CO_2_)_2_(C_4_H_11_NO)_2_] at 25 °C).

Figure 1 shows a perspective view of the Ni^II^ coordination in the title compound; the atom numbering scheme, the interatomic distances and angles are also indicated. The distortion from octahedral symmetry is due to the slight deviation of the internal bite angle of the 2-aminoethanol ligands from 90°, *i.e.* 83.16 (9)° for N1—Ni1—O1^i^, which is similar to that observed in [Ni(CH_3_CO_2_)_2_(C_4_H_11_NO)_2_] (Williams *et al.*, 2001). The title compound is stabilized through inter- and intramolecular O—H···O and N—H···O hydrogen bonds similar to those of other supramolecular crystals of transition metal complexes (Desiraju, 1995, 2007) (Figure 2, Table 1).

The geometry and coordination of the monodentate acetate group in the title compound is comparable to those in [Ni(CH_3_CO_2_)_2_(H_2_O)_4_] (Downie *et al.*, 1971), in [Ni(CH_3_CO_2_)_2_(C_6_H_7_N_3_O)_2_(EtOH)_2_] (Werner *et al.*, 1996), and in [Ni(CH_3_CO_2_)_2_(C_4_H_11_NO)_2_] (Williams *et al.*, 2001). The acetate groups are close to be fully ionized (CH_3_CO_2_^-^); as in a fully ionized acetate, the C–C–O angles (B and C in Figure 3) are about 115.7° and the O–C–O angle is about 126° (A in Figure 3, Table 2) (Williams *et al.*, 2001). The length of the Ni—*O*(acetate) (Table 3), Ni—*O*(non-acetate) (Table 4) and Ni—N bonds (Table 5) in the title compound are comparable to those in similar Ni^II^ complexes, *i.e.* in [Ni(CH_3_CO_2_)_2_(C_4_H_11_NO)_2_] (Williams *et al.*, 2001), [Ni(CH_3_CO_2_)_2_(H_2_O)_4_] (Downie *et al.*, 1971) and [Ni(CH_3_CO_2_)_2_(C_6_H_7_N_3_O)_2_(EtOH)_2_] (Werner *et al.*, 1996).

### Experimental

*Synthesis of title compound.* 5.76 g of nickel (II) acetate tetrahydrate (>=99.0%, Sigma Aldrich) was added to 150 cm^3^ absolute ethanol (>=98, Sigma Aldrich) and mixed with 4.24 g of ethanolamine (>=99.0%, Sigma Aldrich) in a molar ratio of 1:3. The resultant bluish solution was stirred in air for 24 h, paper filtered to remove any insoluble compounds and used for the crystallization of single crystals based on the following procedure: one third of the latter bluish clear solution was removed *via* distillation under vacuum at room temperature. The solution was kept at 5 °C for two weeks to grow the single crystals.

### Refinement

The H atoms of the NH group and OH group were located in a difference map and later restrained to the distance N—H = 0.86 (2) Å and O—H = 0.82 (2) Å. The other H atoms were positioned with idealized geometry using a riding model with C—H = 0.93–0.97 Å. All H atoms were refined with isotropic displacement parameters (set to 1.2 times of the *U*_eq_ of the parent atom).

### Crystal data

**Table:**

[Ni(C_2_H_3_O_2_)_2_(C_2_H_7_NO)_2_]	*F*(000) = 316
*M**_r_* = 298.97	*D*_x_ = 1.544 Mg m^−^^3^
Monoclinic, *P*2_1_/*c*	Mo *K*α radiation, λ = 0.71073 Å
Hall symbol: -P 2ybc	Cell parameters from 1155 reflections
*a* = 5.3284 (5) Å	θ = 2.7–28.0°
*b* = 9.216 (1) Å	µ = 1.53 mm^−^^1^
*c* = 13.133 (2) Å	*T* = 293 K
β = 94.22 (1)°	Rod, light blue
*V* = 643.17 (13) Å^3^	0.16 × 0.08 × 0.06 mm
*Z* = 2	

### Data collection

**Table:**

Oxford Diffraction Xcalibur diffractometer with a Sapphire CCD detector	1314 independent reflections
Radiation source: fine-focus sealed tube	1035 reflections with *I* > 2σ(*I*)
Graphite monochromator	*R*_int_ = 0.022
Rotation method data acquisition using ω and φ scans	θ_max_ = 26.4°, θ_min_ = 3.1°
Absorption correction: multi-scan (*CrysAlis RED*; Oxford Diffraction, 2009)	*h* = −4→6
*T*_min_ = 0.792, *T*_max_ = 0.914	*k* = −11→6
2309 measured reflections	*l* = −16→11

### Refinement

**Table:**

Refinement on *F*^2^	Primary atom site location: structure-invariant direct methods
Least-squares matrix: full	Secondary atom site location: difference Fourier map
*R*[*F*^2^ > 2σ(*F*^2^)] = 0.036	Hydrogen site location: inferred from neighbouring sites
*wR*(*F*^2^) = 0.093	H atoms treated by a mixture of independent and constrained refinement
*S* = 1.03	*w* = 1/[σ^2^(*F*_o_^2^) + (0.0555*P*)^2^ + 0.1361*P*] where *P* = (*F*_o_^2^ + 2*F*_c_^2^)/3
1314 reflections	(Δ/σ)_max_ < 0.001
89 parameters	Δρ_max_ = 0.32 e Å^−^^3^
3 restraints	Δρ_min_ = −0.25 e Å^−^^3^

### Special details

**Table:**

Geometry. All e.s.d.'s (except the e.s.d. in the dihedral angle between two l.s. planes) are estimated using the full covariance matrix. The cell e.s.d.'s are taken into account individually in the estimation of e.s.d.'s in distances, angles and torsion angles; correlations between e.s.d.'s in cell parameters are only used when they are defined by crystal symmetry. An approximate (isotropic) treatment of cell e.s.d.'s is used for estimating e.s.d.'s involving l.s. planes.
Refinement. Refinement of *F*^2^ against ALL reflections. The weighted *R*-factor *wR* and goodness of fit *S* are based on *F*^2^, conventional *R*-factors *R* are based on *F*, with *F* set to zero for negative *F*^2^. The threshold expression of *F*^2^ > σ(*F*^2^) is used only for calculating *R*-factors(gt) *etc*. and is not relevant to the choice of reflections for refinement. *R*-factors based on *F*^2^ are statistically about twice as large as those based on *F*, and *R*- factors based on ALL data will be even larger.

### Fractional atomic coordinates and isotropic or equivalent isotropic displacement parameters (Å^2^)

**Table:**

	*x*	*y*	*z*	*U*_iso_*/*U*_eq_	
C1	−0.0425 (6)	0.1901 (3)	0.0329 (3)	0.0405 (7)	
H1A	−0.1460	0.1586	−0.0269	0.049*	
H1B	−0.0414	0.1133	0.0834	0.049*	
C2	−0.2241 (5)	0.7831 (3)	−0.0038 (2)	0.0373 (7)	
H2A	−0.2846	0.8673	0.0314	0.045*	
H2B	−0.3346	0.7669	−0.0648	0.045*	
C3	0.2827 (5)	0.4706 (3)	0.2084 (2)	0.0344 (7)	
C4	0.5055 (6)	0.5114 (5)	0.2804 (2)	0.0572 (10)	
H4A	0.5577	0.6085	0.2658	0.069*	
H4B	0.6417	0.4455	0.2717	0.069*	
H4C	0.4587	0.5064	0.3495	0.069*	
N1	−0.2255 (4)	0.6560 (3)	0.06274 (18)	0.0304 (5)	
H11N	−0.373 (4)	0.624 (3)	0.069 (2)	0.036*	
H12N	−0.161 (5)	0.681 (3)	0.1224 (16)	0.036*	
O1	−0.1486 (4)	0.3187 (2)	0.07349 (15)	0.0315 (5)	
H1O	−0.077 (5)	0.328 (4)	0.1293 (16)	0.038*	
O2	0.2717 (3)	0.5287 (2)	0.12075 (15)	0.0331 (5)	
O3	0.1255 (4)	0.3817 (3)	0.23830 (16)	0.0475 (6)	
Ni1	0.0000	0.5000	0.0000	0.02502 (18)	

### Atomic displacement parameters (Å^2^)

**Table:**

	*U*^11^	*U*^22^	*U*^33^	*U*^12^	*U*^13^	*U*^23^
C1	0.0472 (17)	0.0281 (15)	0.0470 (17)	−0.0085 (13)	0.0089 (14)	0.0003 (13)
C2	0.0388 (16)	0.0297 (15)	0.0437 (17)	0.0039 (13)	0.0053 (13)	−0.0015 (14)
C3	0.0283 (13)	0.0462 (19)	0.0286 (15)	0.0031 (12)	0.0011 (11)	−0.0047 (12)
C4	0.0442 (18)	0.098 (3)	0.0287 (15)	−0.016 (2)	−0.0052 (14)	0.0002 (19)
N1	0.0269 (11)	0.0343 (13)	0.0303 (13)	−0.0046 (10)	0.0048 (10)	0.0000 (11)
O1	0.0288 (10)	0.0335 (11)	0.0325 (11)	−0.0061 (9)	0.0037 (8)	0.0012 (9)
O2	0.0282 (9)	0.0412 (12)	0.0292 (10)	−0.0055 (8)	−0.0024 (8)	0.0035 (8)
O3	0.0456 (12)	0.0659 (15)	0.0308 (11)	−0.0150 (12)	0.0010 (9)	0.0109 (11)
Ni1	0.0218 (2)	0.0271 (3)	0.0259 (3)	−0.0032 (2)	0.00023 (17)	0.0028 (2)

### Geometric parameters (Å, º)

**Table:**

C1—O1	1.432 (4)	C4—H4B	0.9600
C1—C2^i^	1.518 (4)	C4—H4C	0.9600
C1—H1A	0.9700	N1—Ni1	2.082 (2)
C1—H1B	0.9700	N1—H11N	0.849 (17)
C2—N1	1.462 (4)	N1—H12N	0.863 (17)
C2—C1^i^	1.518 (4)	O1—Ni1	2.1129 (19)
C2—H2A	0.9700	O1—H1O	0.805 (17)
C2—H2B	0.9700	O2—Ni1	2.0841 (19)
C3—O3	1.255 (3)	Ni1—N1^i^	2.082 (2)
C3—O2	1.267 (4)	Ni1—O2^i^	2.0841 (19)
C3—C4	1.510 (4)	Ni1—O1^i^	2.1129 (19)
C4—H4A	0.9600		
			
O1—C1—C2^i^	111.2 (2)	Ni1—N1—H11N	111 (2)
O1—C1—H1A	109.4	C2—N1—H12N	108 (2)
C2^i^—C1—H1A	109.4	Ni1—N1—H12N	110 (2)
O1—C1—H1B	109.4	H11N—N1—H12N	108 (3)
C2^i^—C1—H1B	109.4	C1—O1—Ni1	108.21 (16)
H1A—C1—H1B	108.0	C1—O1—H1O	105 (2)
N1—C2—C1^i^	109.1 (2)	Ni1—O1—H1O	100 (2)
N1—C2—H2A	109.9	C3—O2—Ni1	128.35 (18)
C1^i^—C2—H2A	109.9	N1—Ni1—N1^i^	180.0
N1—C2—H2B	109.9	N1—Ni1—O2^i^	90.00 (9)
C1^i^—C2—H2B	109.9	N1^i^—Ni1—O2^i^	90.00 (9)
H2A—C2—H2B	108.3	N1—Ni1—O2	90.00 (9)
O3—C3—O2	125.0 (3)	N1^i^—Ni1—O2	90.00 (9)
O3—C3—C4	118.6 (3)	O2^i^—Ni1—O2	180.00 (8)
O2—C3—C4	116.4 (3)	N1—Ni1—O1^i^	83.18 (9)
C3—C4—H4A	109.5	N1^i^—Ni1—O1^i^	96.82 (9)
C3—C4—H4B	109.5	O2^i^—Ni1—O1^i^	90.90 (7)
H4A—C4—H4B	109.5	O2—Ni1—O1^i^	89.10 (8)
C3—C4—H4C	109.5	N1—Ni1—O1	96.82 (9)
H4A—C4—H4C	109.5	N1^i^—Ni1—O1	83.18 (9)
H4B—C4—H4C	109.5	O2^i^—Ni1—O1	89.10 (8)
C2—N1—Ni1	106.76 (16)	O2—Ni1—O1	90.90 (7)
C2—N1—H11N	113 (2)	O1^i^—Ni1—O1	180.00 (9)
			
C1^i^—C2—N1—Ni1	−43.2 (3)	C3—O2—Ni1—N1	84.3 (2)
C2^i^—C1—O1—Ni1	32.6 (3)	C3—O2—Ni1—N1^i^	−95.7 (2)
O3—C3—O2—Ni1	0.3 (4)	C3—O2—Ni1—O1^i^	167.5 (2)
C4—C3—O2—Ni1	179.4 (2)	C3—O2—Ni1—O1	−12.5 (2)
C2—N1—Ni1—O2^i^	−70.53 (18)	C1—O1—Ni1—N1	173.11 (17)
C2—N1—Ni1—O2	109.47 (18)	C1—O1—Ni1—N1^i^	−6.89 (17)
C2—N1—Ni1—O1^i^	20.37 (17)	C1—O1—Ni1—O2^i^	83.22 (17)
C2—N1—Ni1—O1	−159.63 (17)	C1—O1—Ni1—O2	−96.78 (17)

Symmetry code: (i) −*x*, −*y*+1, −*z*.

### Hydrogen-bond geometry (Å, º)

**Table:**

*D*—H···*A*	*D*—H	H···*A*	*D*···*A*	*D*—H···*A*
N1—H11*N*···O2^ii^	0.85 (2)	2.24 (2)	3.071 (3)	168 (3)
N1—H12*N*···O3^iii^	0.86 (2)	2.60 (2)	3.352 (3)	146 (3)
O1—H1*O*···O3	0.81 (2)	1.80 (2)	2.587 (3)	166 (3)

Symmetry codes: (ii) *x*−1, *y*, *z*; (iii) −*x*, *y*+1/2, −*z*+1/2.

### Geometry of monodentate acetate groups in different octahedral Ni^II^ complexes (for definitions of bond lengths and angles, please refer to Fig. 3).

**Table:**

Complex	C—O^a^	C—O^b^	C—C	A	B	C	Reference
[Ni(CH_3_CO_2_)_2_(C_2_H_7_NO)_2_]	1.255 (3)	1.267 (4)	1.510 (4)	125.0 (3)	118.6 (3)	116.4 (3)	This work
[Ni(CH_3_CO_2_)_2_(C_4_H_11_NO)_2_]	1.260 (4), 1.249 (4)	1.263 (4)	1.498 (5), 1.504 (5)	124.6 (3), 125.7 (3)	118.0 (3), 117.7 (3)	117.4 (3), 116.6 (3)	Williams *et al.* (2001)
[Ni(CH_3_CO_2_)_2_(H_2_O)_4_]	1.255 (5)	1.272 (5)	1.503 (3)	122.5	119.5	117.9	Downie *et al.* (1971)
[Ni(CH_3_CO_2_)_2_(C_6_H_7_N_3_O)_2_(EtOH)_2_]	1.255 (4)	1.265 (4)	1.511 (5)	124.7 (3)	117.3 (3)	117.9 (3)	Werner *et al.* (1996)

### Ni—O(acetate) bond lengths (Å) and angles (°) in different Ni^II^ complexes.

**Table:**

Complex	Ni—O(acetate)	Reference
[Ni(CH_3_CO_2_)_2_(C_2_H_7_NO)_2_]	2.0841 (19)	This work
[Ni(CH_3_CO_2_)_2_(C_4_H_11_NO)_2_]	2.050 (2), 2.043 (2)	Williams *et al.* (2001)
[Ni(CH_3_CO_2_)_2_(H_2_O)_4_]	2.067 (3)	Downie *et al.* (1971)
[Ni(CH_3_CO_2_)_2_(C_6_H_7_N_3_O)_2_(EtOH)_2_]	2.118 (2)	Werner *et al.* (1996)

### Ni—O(non-acetate ligand) bond lengths (Å) in different Ni^II^ complexes.

**Table:**

Complex	Ni—O(non-acetate ligand)	Reference
[Ni(CH_3_CO_2_)_2_(C_2_H_7_NO)_2_]	Ni—O (C_2_H_7_NO) 2.1129 (19)	This work
[Ni(CH_3_CO_2_)_2_(C_4_H_11_NO)_2_]	Ni—O (C_4_H_11_NO) 2.111 (2), 2.109 (2)	Williams *et al.* (2001)
[Ni(CH_3_CO_2_)_2_(H_2_O)_4_]	Ni—O (H_2_O) 2.048 (4)	Downie *et al.* (1971)
[Ni(C_5_H_8_O_2_)_2_(C_4_H_11_NO)]	Ni—O (C_5_H_8_O_2_) 2.026 (3), 2.013 (4), 2.024 (4), 2.2045 (3); Ni—O (C_4_H_11_NO) 2.111 (4)	Williams *et al.* (2001)

### Ni—N bond lengths (Å) in different Ni^II^ complexes.

**Table:**

Complex	Ni—N (Å)	Reference
[Ni(CH_3_CO_2_)_2_(C_2_H_7_NO)_2_]	Ni—N 2.082 (2)	This work
[Ni(CH_3_CO_2_)_2_(C_4_H_11_NO)_2_]	Ni—N 2.142 (3), 2.145 (3)	Williams *et al.* (2001)
[Ni(C_5_H_8_O_2_)_2_(C_4_H_11_NO)]	Ni—N 2.169 (4)	Williams *et al.* (2001)
[Ni(CH_3_CO_2_)_2_(C_6_H_7_N_3_O)_2_(EtOH)_2_]	Ni—N 2.054 (3), 2.116 (3)	Werner *et al.* (1996)
